# Health, Safety, and Aging in Elderly Farmers in the United States and Beyond: A Scoping Review

**DOI:** 10.1093/geroni/igaf122.2829

**Published:** 2025-12-31

**Authors:** Shuang Li

**Affiliations:** North Carolina Agricultural and Technical State University, Greensboro, North Carolina, United States

## Abstract

Understanding elderly farmers’ health and risk factors is fundamental to preventing agricultural injuries and illnesses as the farmer population ages. Few reviews have focused on how aging impacts farmers’ health and safety. This study reviews scientific literature that characterizes aging farmers’ physical and mental health, work-related risk factors, and potential interventions to support healthy work and aging. Following the framework from Arksey and O’Malley (2003), we conducted a scoping review by exclusively searching MEDLINE (via PubMed), EBSCOhost (via CINAHL), Web of Science (via Clarivate), AGRIS database, and Embase (via Elsevier). Studies that examined older farmers’ health and related risk factors as the primary outcome were included. Eligibility criteria included studies written in English and conducted in the US and globally from 1985-2024. Following PRISMA-ScR (2019) guideline, and findings were synthesized without meta-analysis. Seventy-one studies were included in the final review. Our review indicates that injuries, chronic and occupational diseases, mental health, health risk factors, aging, and retirement considerations have been reported among aging farmers. Major gaps are identified in the study of healthy aging farmers with few studies characterized the trajectory of farmers’ exposure to occupational hazards and adverse health outcomes as they age. A deeper understanding of aging and farmers’ health, such as through longitudinal panel studies, is needed to identify key prevention strategies that keep farmers active and productive until they reach a reasonable retirement age and guidance on societal efforts that would allow older farmers to retire when it makes sense to do so.

